# Multiple novel caliciviruses identified from stoats (Mustela erminea) in the United Kingdom

**DOI:** 10.1099/acmi.0.000813.v4

**Published:** 2024-07-09

**Authors:** Joseph Hinds, Ternenge Apaa, Rhys H. Parry, Amy J. Withers, Laura MacKenzie, Ceri Staley, Joshua Morrison, Malcolm Bennett, Samantha Bremner-Harrison, Elizabeth A. Chadwick, Frank Hailer, Stephen W.R. Harrison, Xavier Lambin, Mathew Loose, Fiona Mathews, Rachael Tarlinton, Adam Blanchard

**Affiliations:** 1 School of Veterinary Medicine and Science, University of Nottingham, Sutton Bonington, UK; 2 Animal and Plant Health Agency, Addlestone Surrey, UK; 3 School of Chemistry and Molecular Biosciences, The University of Queensland, St. Lucia, Australia; 4 School of Biological Sciences, University of Aberdeen, Aberdeen, UK; 5 School of Animal, Rural and Environmental Sciences, Nottingham Trent University, Southwell, UK; 6 Vincent Wildlife Trust, Eastnor, Ledbury, UK; 7 Organisms and Environment, School of Biosciences, Cardiff University, Cardiff, UK; 8 Cardiff University-Institute of Zoology Joint Laboratory for Biocomplexity Research, Beijing, PR China; 9 School of Veterinary Medicine, University of Central Lancashire, Preston, UK; 10 School of Life Sciences, University of Nottingham, Nottingham, UK; 11 School of Life sciences, University of Sussex, Falmer, UK

**Keywords:** calicivirus, metagenomics, *Mustela erminea*, mustelid, stoat, valovirus, vesivirus

## Abstract

The *Caliciviridae* family*,* comprising positive-sense RNA viruses, is characterised by its non-enveloped, small virions, broad host range, and notable tendency for host switching. These viruses are primarily associated with gastroenteric disease, though they can lead to haemorrhagic or respiratory infections. Our study employed a metagenomics analysis of faecal samples from stoats (*Mustela erminea*), identifying two novel calicivirus species, named stoat vesivirus and stoat valovirus. Stoat vesivirus was identified in three samples (ST008, ST006, ST004), exhibiting a genome wide nucleotide identity of approximately 92 %. The complete coding sequences of these samples were 8471 (ST004) and 8322 (ST006) nucleotides in length, respectively. Each comprised three open reading frames (ORF), closely resembling the *Vesivirus* mink calicivirus (China/2/2016), with 70–72 % similarity in ORF1, 61–62 % in ORF2 and 71 % in ORF3. Phylogenetic analysis robustly supported stoat vesivirus as belonging within the *Vesivirus* genus. The second calivicirus (stoat valovirus), detected solely in sample ST008, was 6527 nucleotides in length and with complete coding sequences present. It shared highest similarity with St-Valérien swine virus and marmot norovirus HT16, showing 39.5 and 38.8 % protein identity with ORF1 and 43.3 and 42.9 % for VP1. Stoat valovirus is borderline for meeting the ICTV criteria for a new genus, demonstrating 60 % divergence in ORF1 compared to the other valovirus’, however it clusters basally within the *Valovirus* genus, supporting leaving it included in this genus.

## Data Availability

Novel Stoat calicivirus sequences identified in this study have been deposited to the NCBI GenBank database under accession numbers OR102912-OR102914 and PP066889. Illumina read datasets are available under BioProject accession number PRJNA897822, BioSample accession numbers SAMN3158039, SAMN31580331, SAMN31580344 and SRA accession numbers SRS15672848-50.

## Introduction

*Caliciviridae* (order *Picornavirales*) comprise a diverse family of viruses encompassing 11 known genera. These include mammalian viruses such as *Norovirus, Sapovirus, Lagovirus, Nebovirus, Recovirus, Valovirus* and *Vesivirus*: avian *Bavovirus* and *Nacovirus,* and piscine *Salovirus* and *Minovirus* [1,3]. Caliciviruses are characterised by their small (approximately 27–40 nm in diameter), positive-sense RNA, non-enveloped structure and icosahedral symmetry [4]. The virions are comprised of a major capsid protein, VP1, a minor structural protein, VP2 and a single molecule of RNA which is polyadenylated with the VPg protein covalently linked to its 5′ terminus [1,5, 6].

The genome of calicivirus members varies from 6.4 to 8.3 kb in length and typically includes 2–3 open reading frames (ORF), varying by genus. All caliciviruses produce a polyadenylated sub-genomic RNA coding for the capsid (VP1) and minor basic structural protein VP2, which facilitates enhanced translation of structural genes for virion assembly and packaging [5].

In all caliciviruses, ORF1 encodes for a non-structural polyprotein. This polyprotein undergoes post-transcriptional cleavage by the 3C-like virus protease, resulting in non-structural proteins NS1/2, helicase, NS4, VPg, 3C-like protease, and the viral RNA dependent RNA polymerase. In certain genera like *Bavovirus*, *Lagovirus*, *Minovirus*, *Nacovirus*, *Nebovirus*, *Salovirus*, *Sapovirus* and *Valovirus*, the polyprotein is fused in frame and contains VP1 [3,4]. ORF2 contains the minor structural protein VP2. However, in certain caliciviruses such as vesiviruses and noroviruses, VP1 capsid and VP2 are encoded by ORF2 and ORF3, respectively [2]. The ORF1 and ORF2 regions in vesiviruses are separated by either GC in feline and CCACT in marine caliciviruses [3]. Structurally, VP1 is divided into two domains [7]; the N-terminal shell (S) domain, integral to icosahedral scaffold assembly, and the C-terminal protruding domain (P), forming arching structures on the virion surface. The S domain is highly conserved and the P domain highly variable. This is likely due to the P domain being the site of virus to host cell attachment residues and therefore the determinant of host cell tropism [8,9].

The family *Caliciviridae* are important viruses with frequent discoveries of both novel genus and viral species in recent years [3]. Calicivirus infection can cause significant clinical disease depending on genus and strain; a common example being *Norovirus* infection which can cause a brief gastrointestinal disease with diarrhoea, nausea, vomiting, abdominal pain, myalgias and headaches in humans [10]. Caliciviruses are also a major cause of diarrhoea in cattle and pigs [11]. Rabbit haemorrhagic disease virus (RHDV) (genus *Lagovirus*) causes a lethal form of viral hepatitis in rabbits (*Oryctolagus cuniculus*) with a mortality rate of 70–100 % [11]. It is highly infectious and virulent and has devastated European rabbit populations [12]. There are also rarer manifestations such as hepatitis and encephalitis in mice (*Mus musculus*) [13].

*Vesivirus* infection can also cause a range of other symptoms. Feline calicivirus (FCV) is highly infectious and causes respiratory and oral disease resulting in fever, nasal congestion, ulcers, and lethargy. Extreme cases can cause abortion in pregnant cats, crusting sores, hair loss, chronic gingivostomatitis, fatal pneumonia and liver damage [14]. Vesicular exanthema of swine virus causes disease in swine which is clinically indistinguishable from foot-and-mouth disease, with skin and mucosal vesicles, reproductive failure and mild encephalitis [15]. The *Vesivirus* genus also has a wide and expanding range of hosts such as mink (*Neogale vison*) [16], domestic cats (*Felis catus*) [14], rock rattlesnakes (*Crotalus lepidus*) [17], California sea lions (*Zalophus californianus*) [18] and domestic dogs (*Canis lupus familiaris*) [19].

While noroviruses are well characterised in human infection, vesiviruses are not generally thought of as zoonotic, however there are number of reports cataloguing human infection [16,20,23] with the hom-1 strain of *Vesivirus* identified via accidental exposure of a human host to the San Miguel sea lion virus in a laboratory in 1998 [24]. However interspecies transmission of vesiviruses seems to be a rare event at present with little risk to the human population.

Less is known about the *Valovirus* genus as the only ICTV ratified member is St-Valérien calicivirus or *Saint Valerien virus* (isolate AB90/CAN/2006) which was isolated from asymptomatic pig faeces in the province of Quebec, Canada between 2005 and 2007 [25] and also in Japan [26]. The only other related, tentative *Valovirus* genus member is marmot norovirus, identified in faecal samples of Himalayan marmots (*Marmota himalayana*) in 2013 [27], also with no symptoms of disease reported.

Stoats (*Mustela erminea*) are widespread and native to Eurasia as well as northern portions of North America. They are a carnivorous species that primarily feed on small mammals; however, they are capable of supplementing this diet with a wide range of material such as fruit, eggs, birds and even earthworms when food is scarce [28]. Evidence of stoat calicivirus infections has been suggested prior to this study, with feline calicivirus reactive antibodies detected in several stoats sampled in New Zealand [29] where they are an invasive introduced species. The results strongly indicated the presence of a mustelid calicivirus however no further work was performed and there have been no other stoat caliciviruses identified to date.

This study examined a metagenomics dataset derived from rectal swabs from stoats originally collected for coronavirus screening for the presence of other mammalian viruses, identifying two novel caliciviruses of stoats.

## Methods

### Sample collection and RNA extraction

A total of seven stoat samples were collected during a larger multispecies study on coronaviruses in 402 animals from 14 species [30]. The stoat samples were comprised of both oronasal and rectal swabs of cadavers from lethally trapped wild stoats as part of a pest control programme. Cadavers were within 24 h of death at the time of collection, all animals had separate oronasal and rectal swabs collected, samples were not pooled. The samples were collected in August–September 2021 with ethical approval granted by the University of Nottingham School of Veterinary Medicine and Science (SVMS) Committee for Animal Research and Ethics (CARE) in addition to the University of Sussex Animal Welfare and Ethical Review Board. All stoats were sourced from the Orkney Islands, where they are not native (having been found in 2010) and are the focus of an active eradication programme [31]. Collectors were provided with sampling kits, which included gloves in addition to collection and shipping materials with instructions on collection techniques. Samples were stored in RNAlater at −20°C or directly shipped to the SVMS depending on the equipment available to collectors. RNA extraction was performed on rectal and oronasal swabs using the Macherey-Nagel RNA tissue extraction kit following manufacturers standard protocol.

### High throughput sequencing and genome analysis

RNA sequencing was performed on samples by Novogene UK with the Illumina NovaSeq 6000 platform. Messenger RNA was purified from total RNA using poly-T oligo-attached magnetic beads. After fragmentation, first strand cDNA was synthesized using random hexamer primers followed by second strand cDNA synthesis. In brief library preparation consisted of: end repair, A-tailing, adapter ligation, size selection, amplification, and purification. The library was checked with Qubit and real-time PCR for quantification and bioanalyser for size distribution detection. Quantified libraries were pooled and sequenced on the Illumina platform NovaSeq 6000 S4, according to effective library concentration.

Basecalled fastq files were initially examined using FastQC version (v0.11.9) [32], with adapters and sequence length being trimmed as per default options in fastp (v0.12.4) [32].

Reads were assembled utilising MEGAHIT v1.2.9 [33] using default parameters. The resulting contigs were queried through part of the MetaviralSPAdes pipeline: viralVerify v1.1 [34] followed by viralComplete v1.0 [35]. ViralVerify uses the HMMER database v3.3.2 to check for potential viral sequences followed by viralComplete which scans contigs for complete sequences, producing a list of potential candidates.

Potential novel viral contigs from the MetaviralSPAdes pipeline were extracted and then imported to Geneious Prime version v2022.2 and were manually queried against the NCBI non-redundant nucleotide and protein database using the BLAST+ tool. Open reading frames of calicivirus contigs were predicted using the NCBI ORF finder (https://www.ncbi.nlm.nih.gov/orffinder/). Predicted protein sequences were manually examined for functional viral domains through querying the Pfam database [36] and NCBI’s conserved domain database (CDD) [37] using the CD-Search tool and cross-referencing annotations between existing *Vesivirus* and *Valovirus* sequences as a templates.

For manual validation of the identified calicivirus genomes, trimmed reads were remapped to assembled calicivirus genomes using using Bowtie2 (v2.4.5) [38] under default conditions retaining mapped reads, and manually inspected with Integrated Genomics Viewer (v 2.7.0) [39]. Per position coverage and average coverage was obtained using samtools depth (v1.16.1) and plotted using GraphPad Prism (v10.0.2).

### Phylogenetic analysis

To construct comprehensive phylogenies for the *Caliciviridae* family, including the newly identified stoat caliciviruses species, we initially identified close relatives through BLASTp and BLASTn queries against the NCBI non-redundant database. Additionally, representatives from each *Caliciviridae* genus, as outlined by ICTV demarcation criteria [4], were selected. This resulted in 54 representative sequences for the ORF1 polyprotein and 93 sequences for VP1.

For phylogenetic analysis, we first generated multiple amino acid sequence alignments using the MAFFT-L-INS-I algorithm (v7.515) [40]. Following this, we generated maximum-likelihood phylogenetic inferences based on these alignments using IQ-TREE v2.2.0.3 [41]. Additional parameters used 1000 bootstrap approximations (-B 1000) and SH-aLRT test (--alrt 1000), to test robustness. In constructing both ORF1 and VP1 phylogenetic trees, the best-fit protein substitution model, determined by the Bayesian Information Criterion, was identified as LG+F+R6. The consensus trees were then visualised using FigTree v1.4 (http://tree.bio.ed.ac.uk/software/figtree/).

## Results

Utilising high throughput sequencing, *de novo* assembly and a virus identification pipeline, we identified two novel species of calicivirus from oronasal and rectal samples. Identified sequences were then subject to virus genome annotation and phylogenetic analysis. Metagenome assembled contigs from samples ST008, ST006 and ST004 (all rectal swabs from different animals) showed high pairwise BLASTx identity to the ORF1 protein of *Vesivirus* mink calicivirus, strain China/2/2016 (GenbankID: AXI69355.1, query cover 68 %, identity 76.1 %).

**Fig. 1.**
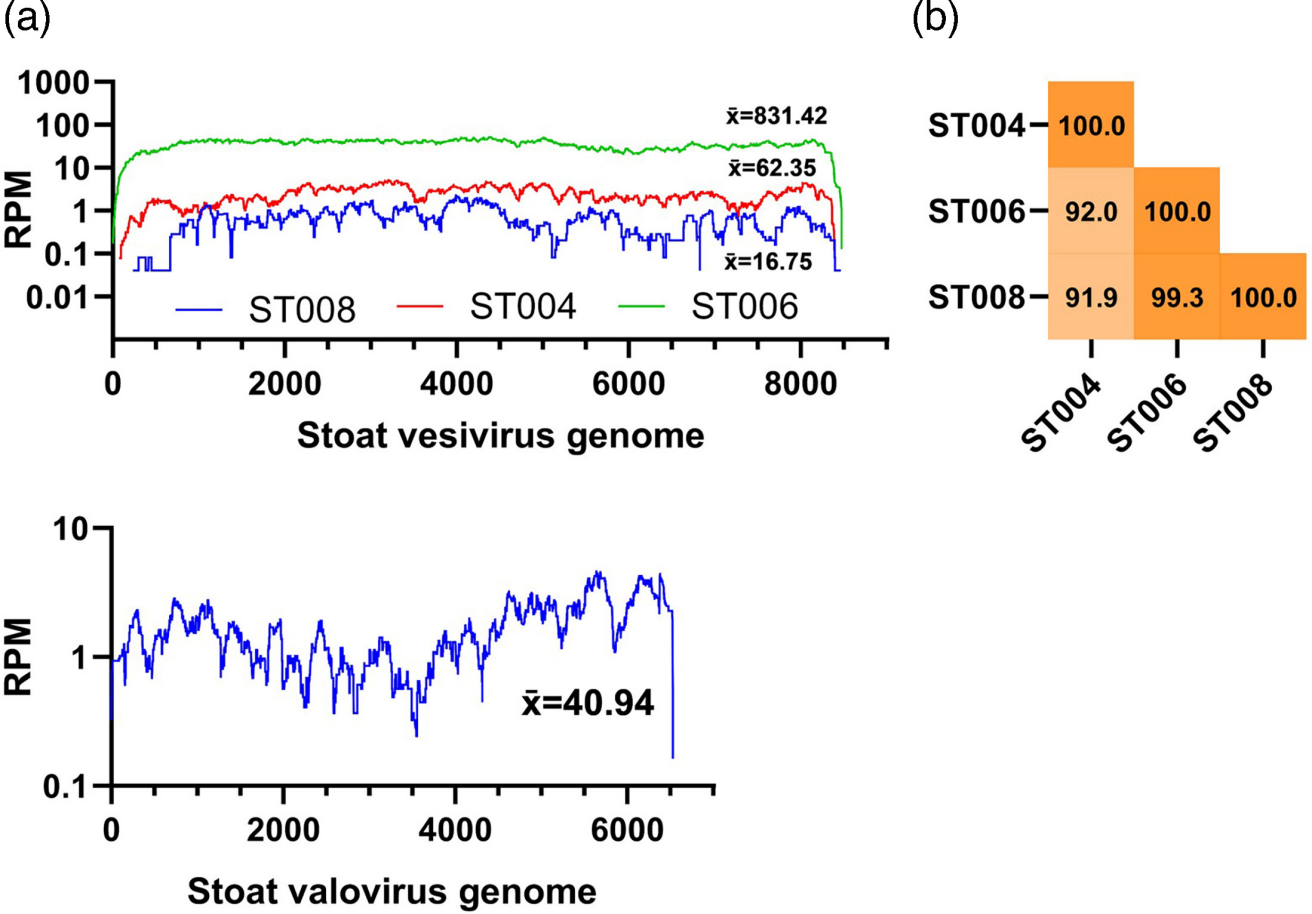
Genome organisation, annotation and coverage of stoat vesivirus and stoat valovirus. (**a**) Coverage for each stoat sample is indicated as mapped reads per million. Genome schematic showing protein domains as coloured boxes, N, Viral polyprotein N-terminal (PF08405); HEL, RNA helicase (PF00910); PRO, Southampton virus-type processing protease C37 (PF05416); Viral RNA-dependent RNA polymerase (PF00680); VP1, calicivirus coat protein (PF00915) and C-terminal (PF08435); and VP2, calicivirus capsid protein (PF03035). (**b**) Pairwise percentage identity of regions with greater than ten-fold depth and mapping quality score of Q20, a conserved 7574nt region for all three strains of the stoat vesivirus genome.

The largest contig, measuring 8 474 nucleotides was selected for re-mapping and reassembly (Fig. 1a). This process yielded complete coverage for samples ST006 and ST004 samples with 830- and 62-fold coverage respectively, and an average 16-fold for sample ST008. Subsequent alignment of these three strains enabled us to perform pairwise identity comparisons across a conserved 7574 nucleotide region. Strains ST008 and ST006 (var1) shared 99.3 % identity, while ST004 (var2) was more divergent sharing between 91.2–92 % identity with the var1 strains (Fig. 1b).

Manual annotation of these contigs revealed three open reading frames absence (no recovery) of the 5′ UTR but 106 nucletiotides (nt) of the 3′ UTR before a polyA tail. The 1947 amino acid ORF1 protein, analysed using BLASTp the NCBI non-redundant virus database, closely matched to the mink vesivirus polyprotein (GenbankIDs: AXI69355.1 and YP_007111844.1), with pairwise amino acid identities of 76.1 %.

Annotation of the ORF1 polyprotein revealed well-characterised viral protein domains and motifs conserved in calicivirus non-structural proteins [1]. The NTPase/helicase motif GPPGCGKT was located at residues 637–644 within the predicted Pfam RNA helicase domain (PF00910, E-value: 4.4e-24). The cysteine protease motif GDCGLP was located at residues 1374–1379 within the C24 endopeptidase cysteine protease domain (Pfam ID: PF03510, E-value: 1.6e-28). Additionally, the viral genome-linked protein domain (VPg) is covalently linked to the 5′ terminal of the genomic RNA (Pfam ID: PF20915, E-value: 4.1e-23). The RNA-dependent RNA polymerase (RdRp) domain motifs KDEL, GLPSG and YGDD were present at residues 1590–1593, 1722–1726 and 1770–1773 respectively, within the Pfam RdRP family (PFam ID: PF00680, E-value: 3.6e-117).

The second ORF encodes a 703 amino acid protein which is most closely related to mink vesivirus, ORF2 with similarities ranging between 61.8 and 62.4 % (Genbank ID: AXI69356.1, YP_007111845.1). ORF2 contains a calicivirus coat protein domain (PfamID: PF00915, E-value: 1.4e-110) between residues 167–442. The third ORF3, encoding a 136aa protein, overlaps with ORF2 by a nucleotide (AT**A****TG**). This overlap is consistent with that of ORF3 of the mink calicivirus strain Mink/China/2/2016 (GenbankID:MF677852.1). Despite incorrect annotation of the mink calicivirus on NCBI, ORF3 shares 79.41 % similarity to the 136aa product encoded by ORF 7896–8303 of the mink calcivirus genome. Annotation of the protein sequence by Pfam protein domain analysis shows that ORF3 encodes a *Vesivirus* VP2 protein domain (PF05332, E-value: 1.3e-11) between residues 15–129.

The second calicivirus contig, measuring 6 527 nt, was identified exclusively in the ST008 sample suggesting a mixed infection with two different caliciviruses. BLASTx analysis of the contig against the NCBI virus non-redundant database revealed the closest pairwise identity with the polyprotein of St-Valérien swine virus strain NC-WGP93C/USA/2009 (Genus *Valovirus*) (GenbankID: ADG27878.1, Identity: 40.2 %) and marmot norovirus (GenbankID: YP_009552830.1, query cover: 90 %, identity 38.8 %) suggesting tentative assignment to the *Valovirus* genus. Remapping reads to this contig demonstrated a coverage of 40-fold (Fig. 1c) of the stoat valovirus. Annotation of the genome organisation and structure revealed two open reading frames, encoding 1995 amino acids and 157 amino acids respectively. The genome also possesses a 5′ UTR of 56 nt, a 14 nt-long UTR, and a poly (A) tail. The genome size and UTRs are similar to the St-Valérien-like valovirus which has a 5′ UTR of 10 nt and a has a 16 nt 3′ UTR before the start of the polyA tail.

ORF1 was manually annotated to identify several virus protein domains. These included a calicivirus viral polyprotein N-terminal domain from positions 15–198 (Pfam domain: PF08405, E-value:1.9e-07), an RNA helicase domain from 368 to 466 (Pfam domain: PF00910, E-value: 1e-25), a weak peptidase C37 hit from 737 to 824 (Pfam domain: PF05416, E-value: 0.05), and viral RNA-dependent RNA polymerase between 1041–1441 (Pfam: PF00680, E-value: 7.5e-50). Consistent with members of the *Valovirus* genus ORF1 encodes a calicivirus coat protein domain between positions 1483 and 1750 (PfamID: PF00915, E-value: 9.1e-47), and a calicivirus coat protein C-terminal domain between positions 1876 and 1993 (PfamID: PF08435, E-value: 7.2e-07). The second ORF, a 157 amino acid protein, overlaps with ORF1 by a nucleotide (AG**A****TG**), and shares the closest identity to the minor basic structural protein VP2 of St-Valérien swine virus strain (NC-WGP93C/USA/2009) (GenbankID: ADG27879.1) with 42.17 % identity . This protein contains a weak calicivirus putative capsid protein domain from positions 11–120 (PfamID: PF03035, E-value: 0.084).

**Fig. 2.**
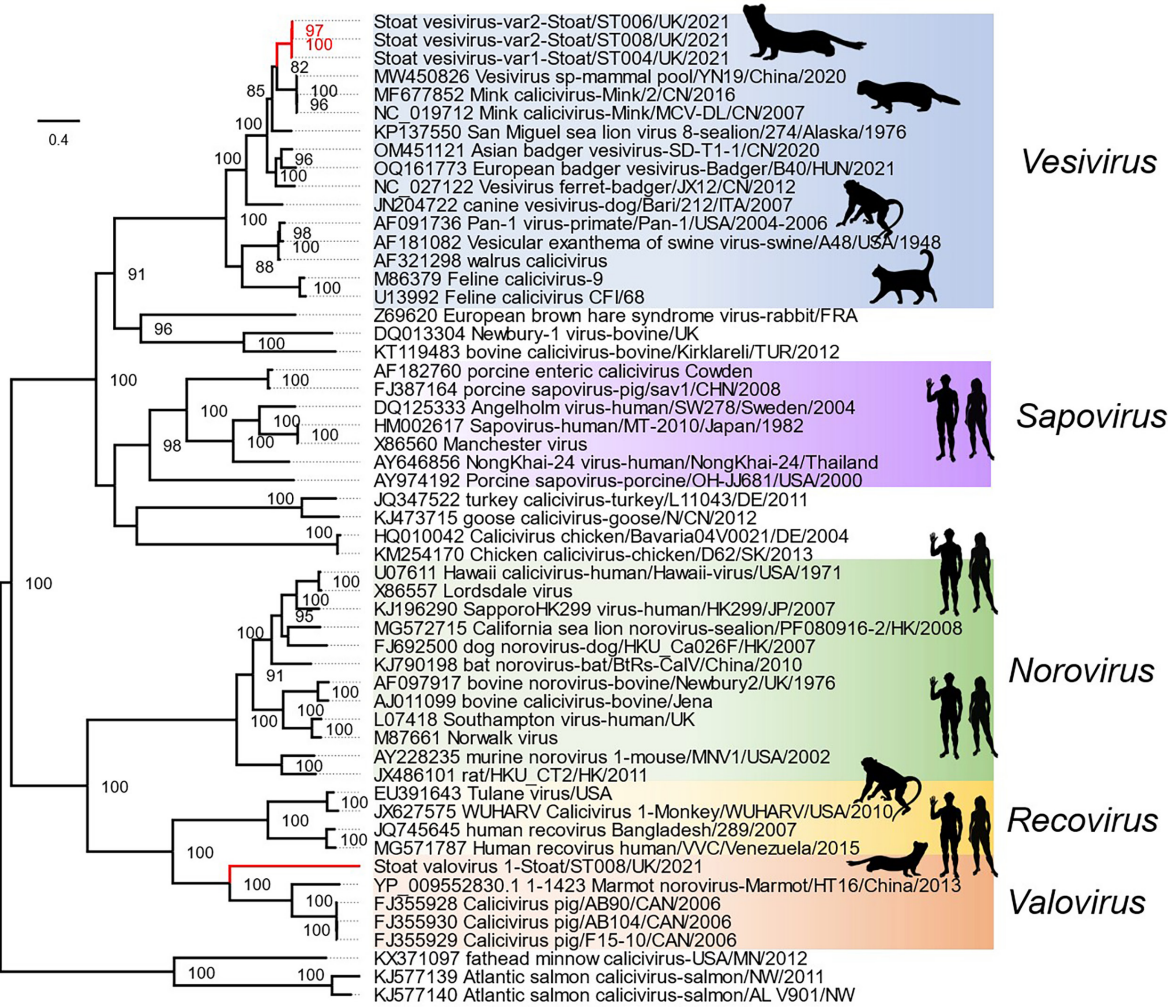
Phylogenetic analysis of stoat vesivirus and stoat valovirus within the *Caliciviridae* family. Maximum likelihood tree of the polyprotein, excluding VP2 in fused polyprotein genera. The tree is midpoint rooted, and genera with ratified calicivirus species are indicated and coloured as per the *Caliciviridae* ICTV Virus Taxonomy Profile 2019. Viruses identified in this study are denoted by a red lines and pictogram of selected host species. The scale bar indicates the number of amino acid substitutions per site, and the protein substitution model was LG+F+R6 chosen according to BIC.

Maximum-likelihood phylogenetic analysis was conducted on the *Caliciviridae* family, incorporating the two novel stoat caliciviruses identified in this study. This analysis included full-length, aligned amino acid sequences for ORF1 (Fig. 2) and ORF2/VP2 (Fig. S1, available in the online Supplementary Material). The resulting phylogeny aligns with the overall topology of the *Caliciviridae* family from other studies [20]. In our phylogenetic tree, stoat vesivirus forms a distinct cluster with vesiviruses identified from other mustelid species, including the European badger (*Meles meles*) [3], Asian badger (*Meles leucurus*) [42], ferret badger [1] (*Melogale moschata*), mink [43], and a mixed pool of small mammals including *Paguma larvata* (masked palm civet), *Callosciurus erythraeus* (Pallas’s squirrel), *Prionailurus bengalensis* (leopard cat) and *Mephitis* sp. (skunk) [44]. The vesiviruses collectively form a monophyletic cluster within the *Caliciviridae*, with mustelid sequences being genetically distinct from those in canines and walrus and clustering separately from a cluster of vesiviruses found in primates, cats, pigs, walruses, and sea lions.

The phylogenetic analysis of the ST008 calicivirus indicates that the stoat valovirus branches off first among the analysed *Valovirus* genus representatives (Fig. 2). When comparing the polyprotein of stoat valovirus with those of the marmot and swine valoviruses, the genetic distance is at the borderline of the criteria for demarcation of a novel genus, typically defined as greater than 60 % amino acid divergence in ORF1. Nevertheless the stoat valovirus forms a monophyletic cluster with the other members of the *Valovirus* genus, which could also support its classification within this genus.

## Discussion

The identification of two divergent caliciviruses, specifically a *Vesivirus* and a *Valovirus*, in stoats aligns with existing knowledge about the *Caliciviridae* family, known for its prevalence across a diverse range of vertebrate hosts. The discovery is unsurprising given the previous detection of vesiviruses in related mustelid species, such as mink [16], norovirus species from the marmot and a previous report of antibodies to vesiviruses (feline calicivirus) present in stoat samples [29]. This virus is part of a group of viruses that appear to be quite mustelid specific with the most closely related viruses found in other mustelids (mink, ferret badgers and Asian badgers). It is likely that further viruses of this group will be identified with greater sampling intensity of this group of animals. Indeed, there are recent reports of caliciviruses in European badgers (*Meles meles*) [3] though sequences for these isolates are not yet publically available.

The pathogenicity of these viruses in their hosts remains unclear. Previous studies speculated that a vesivirus might be linked to respiratory pathology seen in the New Zealand animals [29], and a similar mink vesivirus sequence was originally isolated as part of an investigation into a diarrhoea outbreak in mink [44] which would be consistent with symptoms of *Vesivirus* infection present in other species. Another recent study also identified mink calicivirus in response to reports of infection at a mink farm causing diarrhoea [16]. The ferret badger sequence which is a close relative of the stoat sequence was identified in intestinal samples, though no symptoms were identified in the animals tested [1]. Caliciviruses commonly cause gastrointestinal disease in many species (diarrhoea) including bovine, human and canine [11,19, 20], but other clinical manifestations include vesicular disease in skin (pigs) [15] and mucosal membranes (cats), upper respiratory tract disease (cats) [14] and haemorrhagic fever (rabbits) [45]. Rarer manifestations include hepatitis, encephalitis (mice) and abortions [13].

The stoat population from which these viruses were identified represents a recently established, bottlenecked island population [31], which may not be representative of the wider prevalence or impact of these viruses on mainland animals. Intriguingly, this population also showed a high detection rate of a novel *Minacovirus* (genus *Alphacoronavirus*) [30]. This population may not be very genetically diverse in their MHC allele complement inhibiting their ability to respond to some pathogens. The detection of a *Vesivirus* in a costal location is interesting due to the related viruses being present in many marine mammals and fish [46]. Stoats are known to scavenge for food and eat birds [28] and may acquire infection through consumption of marine animals, as demonstrated in white terns (*Gygis alba*) [47]. The infection of pigs with vesicular exanthema of swine virus (VESV) in the USA was speculated to have come from the use of waste seafood to feed pigs or minks with related viruses from marine strains (fish and marine mammals) [46]. VESV is a curious disease outbreak only seen in pigs in the United States between 1932 and eradicated in 1959 with its aetiology never fully explained.

Though this possibility exists, the genetic distance demonstrated through phylogenetic analysis appears to show significant diversity from vesiviruses present in ocean species or VESV, indicating these mink viruses have come from a different source. Presence of this novel *Vesivirus* in a significant number of samples (3/4) presents the possibility of widespread disease in the bottlenecked population. A further study on mainland populations should be performed to ascertain if this virus is present and to determine its prevalence.

The cross-species transmission potential of these viruses remains uncertain. Vesiviruses and valoviruses are found across a diverse range of species. The sea lion isolates of vesiviruses have been known to infect laboratory workers and cell lines [24] while other members of this group have been identified as cell line contaminants of hamster cells [48]. Stoats however provide a very low risk of transmission to human populations due to lack of close contact situations.

Given the relatively close genetic relationship between the *Valovirus* genus and the *Recovirus* calicivirus genus, the latter which contains viruses that infect primates and humans [49,50], larger comprehensive epidemiologic studies are needed. These studies should integrate both genetic and serologic screening to gain further insight into the distribution and pathogenic potential of *Valovirus* viruses. Previous studies have reported a high prevalence of virus-neutralizing antibodies against a closely related *Recovirus* Tulane virus, in serum samples from animal caretakers [51].

In conclusion, we have identified novel lineages of *Vesivirus* and *Valovirus* from stoat samples. Genomic and phylogenetic analysis has confirmed the taxonomy of these viruses within their respective genera*,* and their close relation to previously identified viruses in minks, marmots, badgers, and ferrets.

## supplementary material

10.1099/acmi.0.000813.v4 Uncited Supplementary Material 1.

10.1099/acmi.0.000813.v4 Uncited Supplementary Material 2.

## Abbreviations

NS: non structural protein (numbered)
ORF: open reading frame
P: protusion domain
RDRP: RNA-dependent RNA polymerase
S: shell domain
UTR: untranslated region
VP1: major capsid protein 1
VP2: major capsid protein 2
VpG: viral protein genome-linked
