# Supplementary material for: Multiple novel caliciviruses identified from stoats (Mustela erminea) in the United Kingdom
Source: Access Microbiol. 2024 Jul 9;6(7):000813.v4. doi: 10.1099/acmi.0.000813.v4 (PMC11316584; doi:10.1099/acmi.0.000813.v4)
Supplement: Uncited Supplementary Material 1. [file acmi-6-00813-s001.pdf]

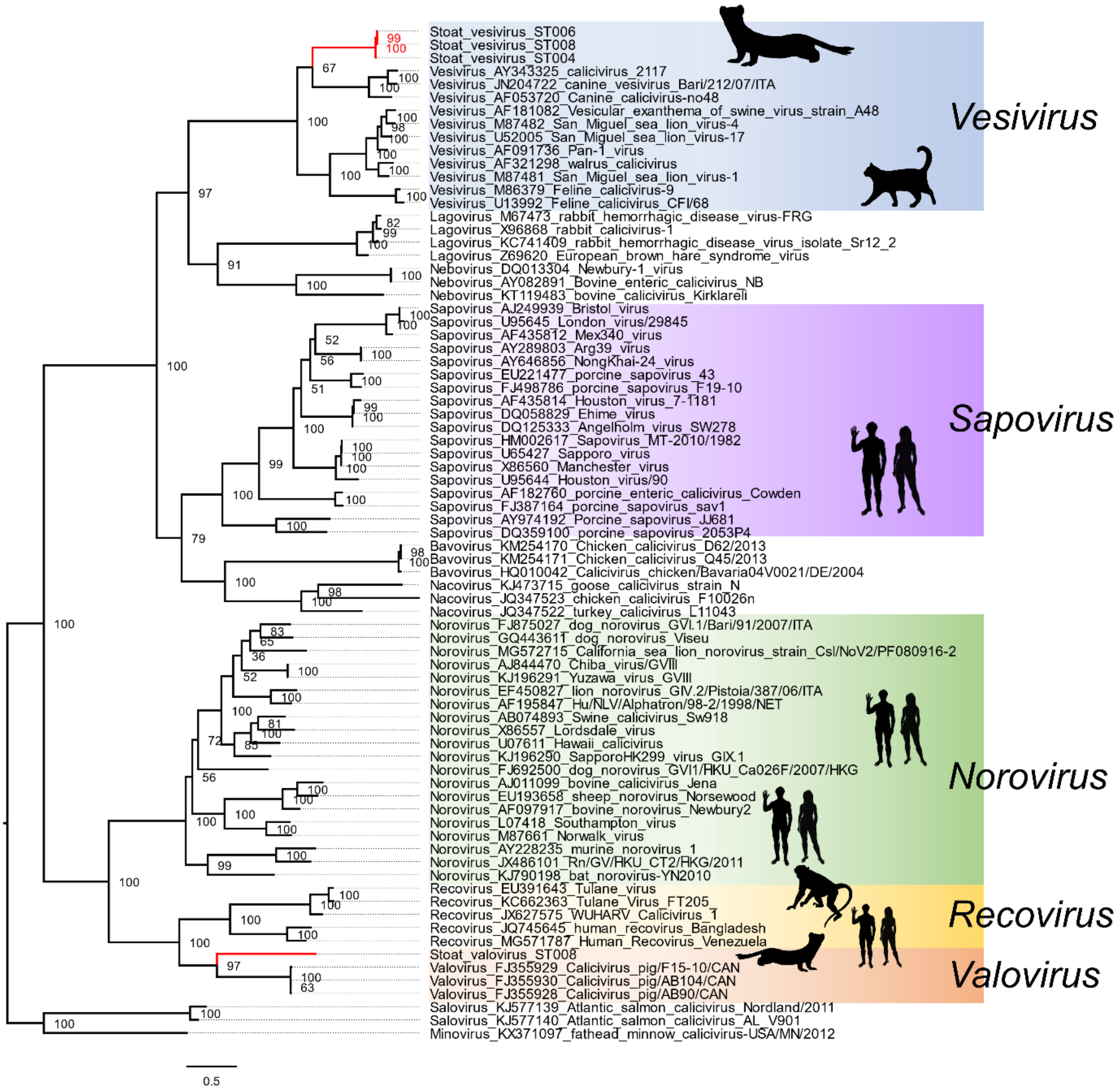

Figure S1: Maximum likelihood phylogenetic analysis of Stoat vesivirus and Stoat valovirus VP1 protein within the *Caliciviridae* family using the ICTV Sequence Calicivirus VP1 resource alignment file (Available from: https://ictv.global/sites/default/files/inline-images/OPSR.Calici.Fig4.alignment.fas). The tree is rooted on the *Salovirus*/*Minovirus* genus members. Selected *Calicivirus* genera species are indicated and coloured as per the *Caliciviridae* ICTV Virus Taxonomy Profile 2019, and genera are on each node. Viruses identified in this study are denoted by a red line and a pictogram of selected host species. The scale bar indicates the number of amino acid substitutions per site, and protein substitution model was LG+F+R6 chosen according to BIC.

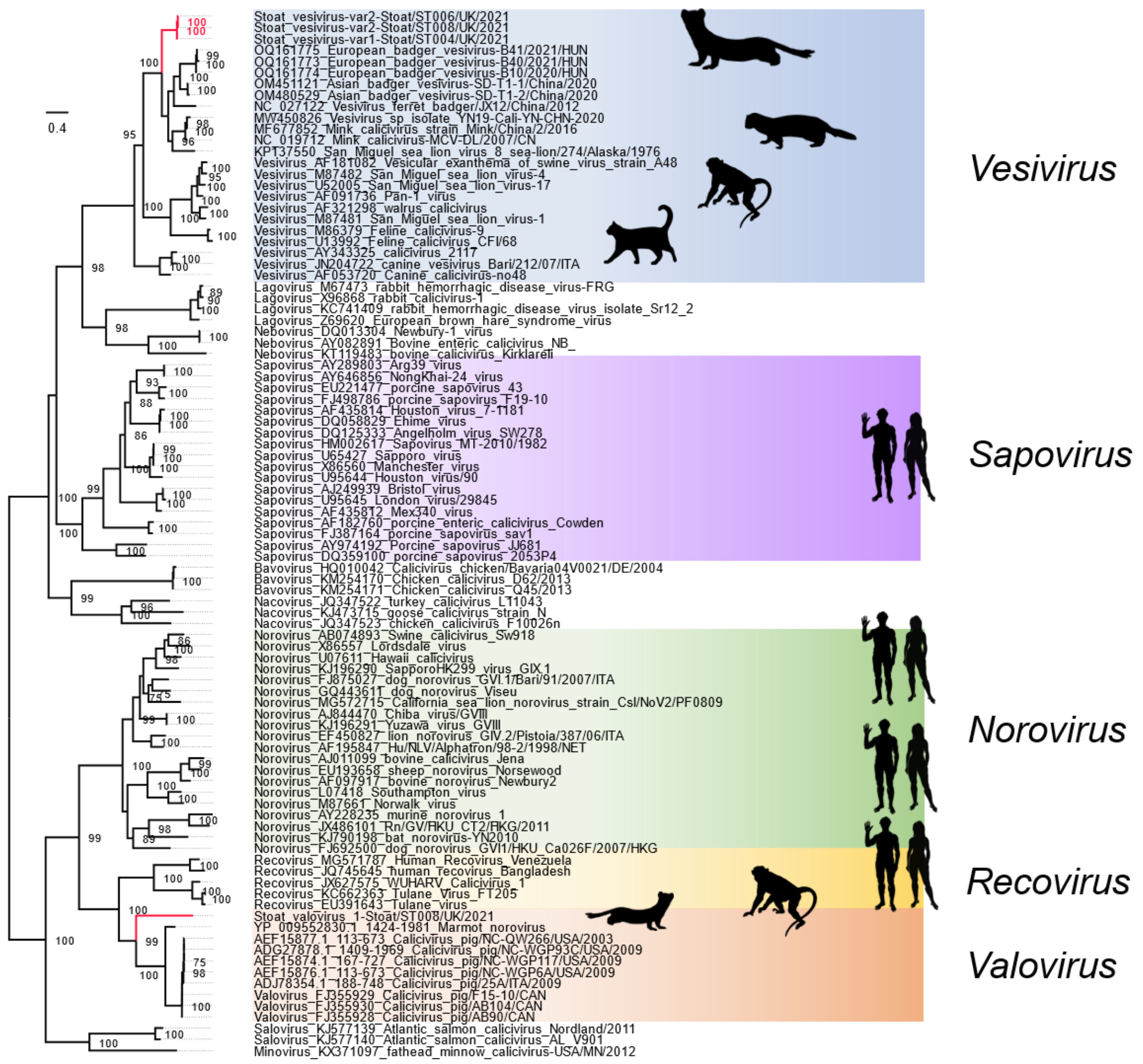

Figure S2: Maximum likelihood phylogenetic analysis of Stoat vesivirus and Stoat valovirus ORF2/VP2 protein within the *Caliciviridae* family. The tree is midpoint rooted, and genera with selected Calicivirus genera are coloured as per the *Caliciviridae* ICTV Virus Taxonomy Profile 2019. Viruses identified in this study are denoted by a red line and a pictogram of selected host species. The scale bar indicates the number of amino acid substitutions per site, and the protein substitution model was LG+F+R6 chosen according to BIC.
