# Supplementary material for: Multiple novel caliciviruses identified from stoats (Mustela erminea) in the United Kingdom
Source: Access Microbiol. 2024 Jul 9;6(7):000813.v4. doi: 10.1099/acmi.0.000813.v4 (PMC11316584; doi:10.1099/acmi.0.000813.v4)

Supplementary information:

Sequences used (accession numbers):

AB070225

AF053720

AF091736

AF182760

AJ011099

AY032605

AY082891

AY343325

AY772538

M87661

DQ013304

DQ285629

EF193004

EF195384

EU391643

FCU13992

FJ355928

FJ355930

GQ475301

GQ475302

GU592498

JX047864

JX847605

KJ701554

KY114613

MF677852

NC004541

NC004542

NC019712

NC027122

NC034444

OM480529

M67473

M87482

L07418

U76874

Z69620

ORF1 phylogenetic distance tree:

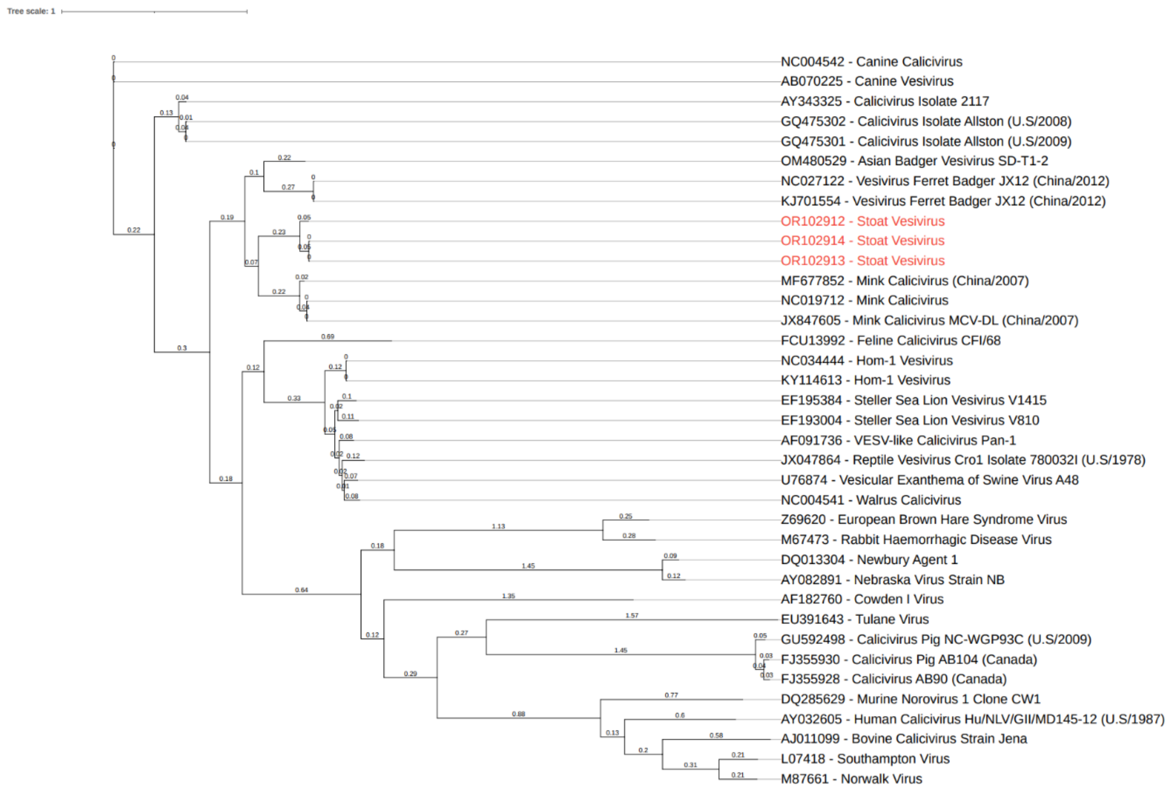

ORF2 phylogenetic distance tree:

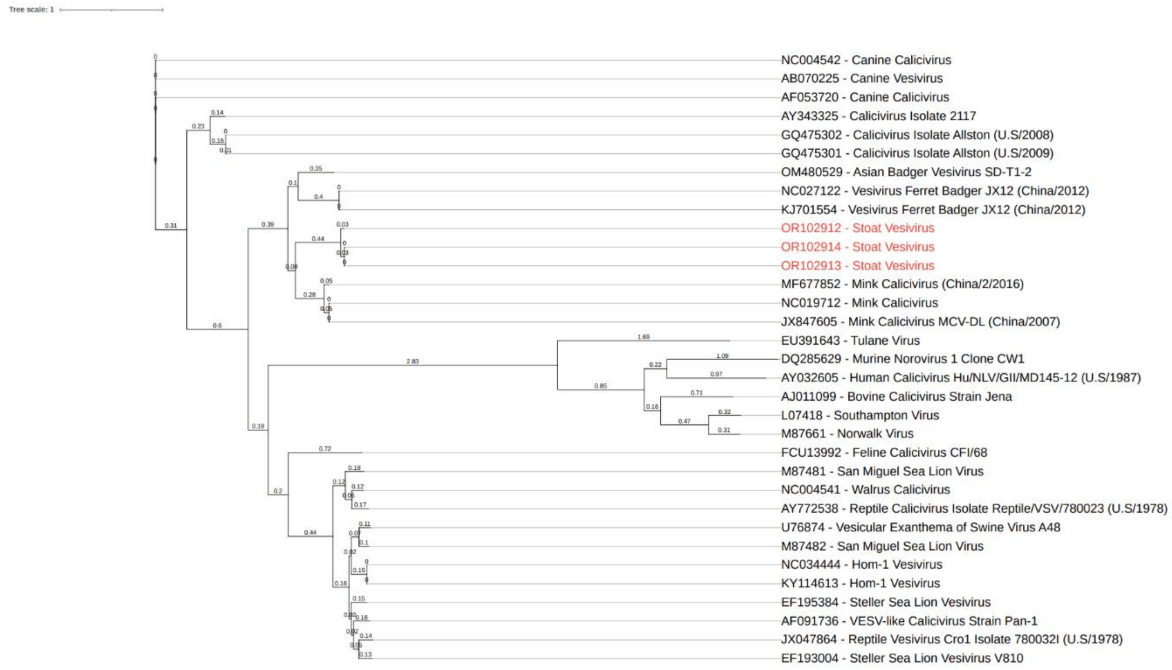

ORF3 phylogenetic distance tree:

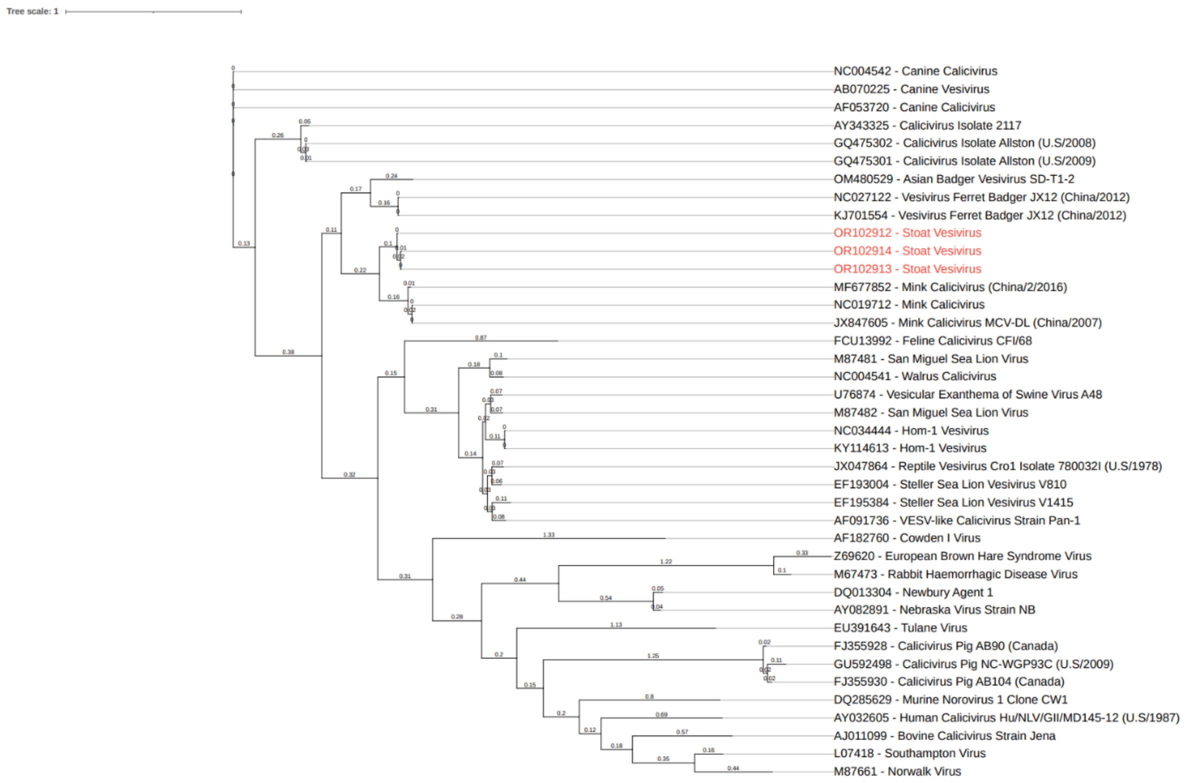

Supplement: Uncited Supplementary Material 2. [file acmi-6-00813-s002.pdf]
